# Childhood Tuberculosis in a Sub-Saharan Tertiary Facility: Epidemiology and Factors Associated with Treatment Outcome

**DOI:** 10.1371/journal.pone.0153914

**Published:** 2016-04-21

**Authors:** Loukia Aketi, Zacharie Kashongwe, Christian Kinsiona, Serge Bisuta Fueza, Jack Kokolomami, Grace Bolie, Paul Lumbala, Joseph Shiku Diayisu

**Affiliations:** 1 Department of Pediatrics, University Hospital of Kinshasa, Kinshasa, Democratic Republic of Congo; 2 Department of Internal Medicine, University Hospital of Kinshasa, Kinshasa, Democratic Republic of Congo; 3 National Tuberculosis Program, Kinshasa, Democratic Republic of Congo; 4 Epidemiology and Biostatistics Department, Public Health School at the University of Kinshasa, Kinshasa, Democratic Republic of Congo; St. Petersburg Pasteur Institute, RUSSIAN FEDERATION

## Abstract

Childhood tuberculosis (TB) is a diagnostic challenge in developing countries, and patient outcome can be influenced by certain factors. We report the disease course, clinical profile and factors associated with treatment outcome in a tertiary facility of Kinshasa. Documentary and analytical studies were conducted using clinical and exploratory data for children aged up to 15 years who were admitted to the University Clinics of Kinshasa for TB. Data are presented as frequencies and averages, and binary and logistic regression analyses were performed. Of 283 children with TB, 82 (29.0%) had smear-negative TB, 40 (14.1%) had smear-positive TB, 159 (56.1%) had extra-pulmonary TB (EPTB), 2 (0.7%) had multidrug-resistant TB (MDR-TB), 167 (59.0%) completed treatment, 30 (10.6%) were cured, 7 (2.5%) failed treatment, 4 (1.4%) died, 55 (19.4%) were transferred to health centers nearest their home, and 20 (7.0%) were defaulters. In the binary analysis, reported TB contacts (p = 0.048), type of TB (p = 0.000), HIV status (p = 0.050), Ziehl-Nielsen test result (p = 0.000), Lowenstein culture (p = 0.004) and chest X-ray (p = 0.057) were associated with outcome. In the logistic regression, none of these factors was a significant predictor of outcome. Tertiary level care facilities must improve the diagnosis and care of patients with childhood TB, which justifies the development of alternative diagnostic techniques and the assessment of other factors that potentially affect outcome.

## Introduction

The Democratic Republic of Congo (DRC) is one of the countries most affected by tuberculosis (TB), with 116,894 cases reported in 2014; it is 10^th^ in the world and 3^rd^ in Africa in terms of TB burden [1]. The DRC shares 80% of the worldwide TB burden with 21 other countries. According to World Health Organization (WHO) statistics, the incidence of TB in the DRC was 325 [295–356] cases per 100,000 inhabitants in 2014. The estimated number of children under 15 years of age with a positive microscopy smear was approximately 3,438 [1]; however, this number was likely underestimated because of diagnostic challenges [2].

In developing countries, the management of childhood TB is challenging [3]. Its morbidity and mortality remain high, making it one of the three primary poverty-related infectious diseases [4, 5, 6]. The difficulties in diagnosing children lead to a low rate of case identification [7, 8], and the childhood TB burden reflects the extent of TB among adults. In countries with highly limited resources, TB control depends not only on epidemiological factors but also on clinical and therapeutic factors [9]. Successful disease control is dependent on improved coverage of TB services, a fully functional referral system from facilities to the community, regular evaluation of activities, and the development of alternative diagnostics [10].

In the DRC, little is known about the epidemiology, clinical treatment or outcome of childhood TB. This study aimed to contribute to childhood TB management by investigating whether certain factors are associated with poor treatment outcomes among children living in a developing country who initiate TB treatment in a tertiary hospital presumed to correctly apply the principles of TB treatment. The objective of this study was to report the disease course, clinical profile, and factors associated with therapeutic outcome for children with TB at the University Clinics of Kinshasa (UCK).

## Materials and Methods

This is a retrospective analysis of data collected from January 2005 to December 2011 on patients suffering from TB who were admitted to UCK. This tertiary level health care facility is located on the outskirts of Kinshasa and is surrounded by suburbs in which most of the patients live. The UCK also receives cases transferred from primary or secondary level hospitals all around the city and country. According to the national guidelines for TB, after diagnosis, patients suffering from TB are referred to the nearest health center for treatment. We enrolled all children aged 0 to 15 years who were admitted to the hospital for any form of TB during the study period. These patients had TB that was confirmed bacteriologically or diagnosed by a clinician [11]. The register of patients contained patient forms and reports, and all the data were collected on paper. We divided the included patients into 4 age groups (0 to 2 years, 3 to 6 years, 7 to 10 years, and 11 to 15 years). The demographic (age, gender, reported TB contact, residence and household size), clinical (weight and clinical manifestations) and paraclinical (sputum or gastric liquid microbiology, HIV status, radiography results and histopathology results) data were recorded. The nutritional status of each patient under 10 years old (reference data for children over 10 years old were not available), especially the weight-for-age z-score, was analyzed according to the WHO Multicenter Growth Reference Study [12] using WHO Anthro and Anthro Plus software version 3.2.2, 2011. Malnutrition was characterized based on WHO definitions: no malnutrition, z-score between -2 and 2; moderate malnutrition, z-score between -2 and -3; and severe malnutrition, z-score <-3.

**Fig 1:**
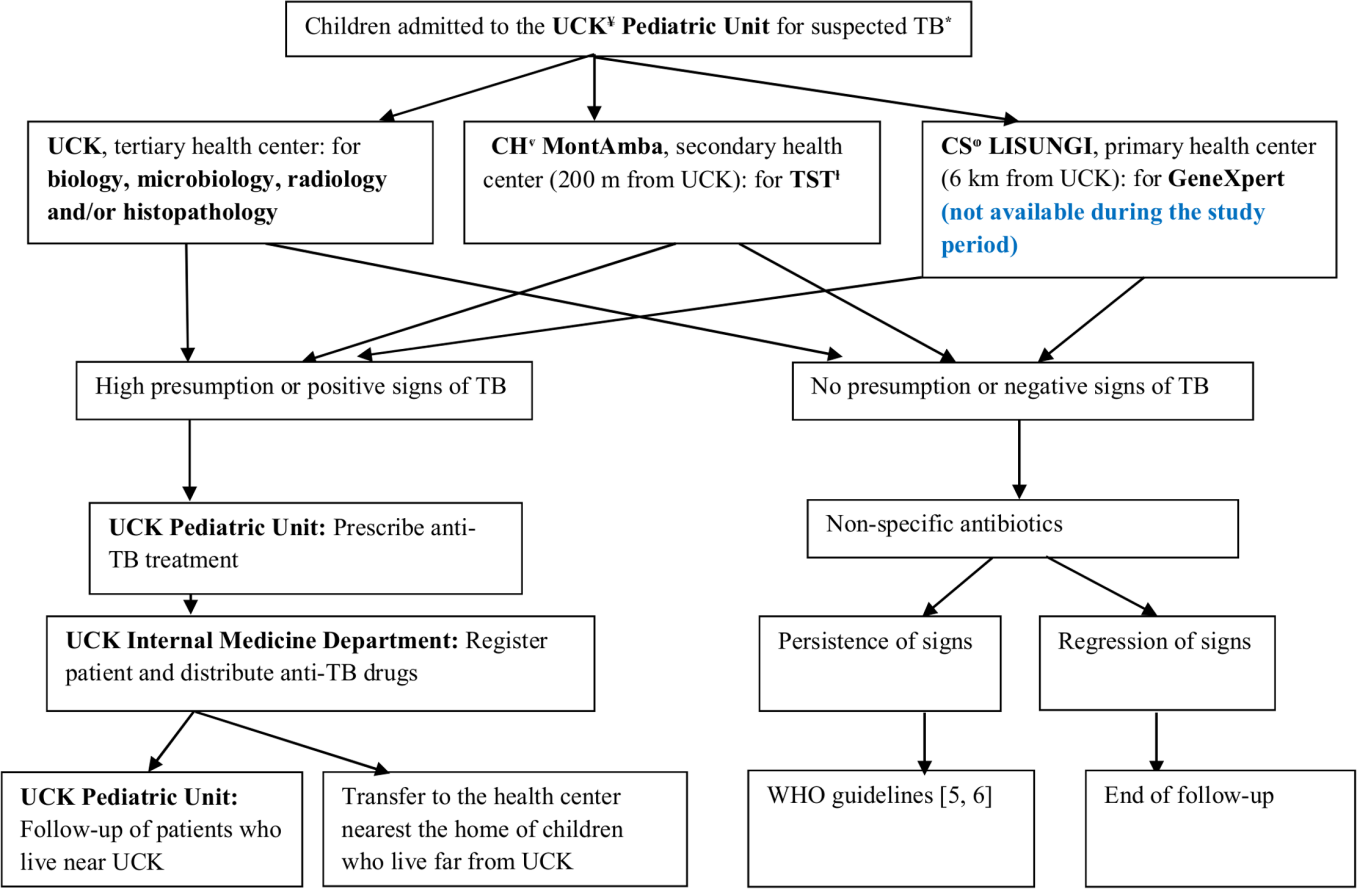
Flow-chart of children admitted to the pediatric unit for TB.

However, since the generation of our patient cohort, TB diagnosis has improved because of clinical elements associated with one or several positive elements, namely, the Keith-Edwards score, the tuberculin skin test (TST), AFB coloration (of sputum for children older than 6 years, of gastric fluid for those less than 6 years or of any other biological fluid or effusion at any age), chest X-ray and Lowenstein culture (with antibiogram). Histopathological testing of adenitis was performed in a few patients. The Gene Xpert was not yet available in the DRC during the study period. It has been available since 2012 in some primary health centers, such as Lisungi Health Center, which is the nearest center to UCK that has this platform (Fig 1).

In addition, TB was diagnosed according to the national program guidelines [13] and the WHO diagnostic approach [14] using one of the following definitions: positive pulmonary TB sputum smear (PTB+); negative pulmonary TB sputum smear (PTB-); extra-pulmonary TB (EPTB); or drug-resistant TB (DR-TB). We considered all pulmonary cases with lesions in the lung parenchyma and extrapulmonary cases with lesions outside the lung parenchyma [15]. Children who had both pulmonary and EPTB were classified as having pulmonary TB [16]. Common forms of EPTB were diagnosed based on a positive result on an appropriate test: peripheral lymphadenitis TB: lymph node biopsy or fine needle aspiration; miliary TB: chest X-ray; TB meningitis: lumbar puncture with cerebrospinal fluid analysis or cerebral computed tomography; pleural effusion TB: chest X-ray and pleural tap; abdominal TB: abdominal ultrasound and ascitic tap; osteoarticular TB: osteoarticular X-ray, joint tap or synovial biopsy; and pericardial TB: pericardial ultrasound and pericardial tap [16].

Concerning treatment, children were classified into one of 4 therapeutic categories [13, 16]: category 1: any new cases of PTB+ or PTB- with extensive parenchymal involvement, severe forms of extrapulmonary TB and severe concomitant HIV infection; category 2: any previously treated cases of PTB+, including relapse, treatment after interruption and treatment failure; category 3: any cases of uncomplicated TB, such as new smear-negative pulmonary TB (other than those in category 1) and less severe forms of extrapulmonary TB; and category 4: chronic TB (any cases of treatment failure or persistence of TB signs after adequate retreatment) and multidrug-resistant TB (MDR-TB).

The treatment regimen [13] for categories 1 and 3 was 2 months of treatment (intensive phase) with rifampin (R), isoniazid (H), ethambutol (E) and pyrazinamide (Z) (2RHEZ) followed by 4 months of treatment (continuation phase) with rifampin and isoniazid (4RH); in cases of TB meningitis, ethambutol was replaced with streptomycin (S) during the intensive phase. For category 2, the treatment regime was 2 months of isoniazid, rifampin, ethambutol, pyrazinamide and streptomycin (2SRHEZ), followed by 1 month of RHEZ (1RHEZ) and 5 months of RHE (5RHE). For category 4, the treatment regimen was 6 months of kanamycin (K), ofloxacin (O), prothionamide (P), ethambutol (E), pyrazinamide (Z), and cycloserine (C) (6KOPEZC), followed by 18 months of the same combination but without injectable agents. Currently, new guidelines according to the new WHO recommendations are in press at the National Program for Tuberculosis and Leprosy (NPTL).

After diagnosis, patients were sent to the TB unit of the internal medicine department for TB drug administration. Hospitalized patients and children whose parents were absent or had a limited comprehension of the instructions received directly observed therapy, and the other patients underwent self-supervised therapy. Parents or tutors received advice on disease management and treatment adherence, and the patients received aliquots of anti-TB drugs so that adherence could be controlled at each visit, for example, by counting the remaining drugs in the pack. At the same time, pediatricians could perform clinical monitoring and manage side effects (Fig 1).

As operational definitions, all patients who were declared “cured” and/or “completed treatment” were termed a “treatment success”, and “poor treatment outcome” was used for all patients who defaulted, died, or experienced treatment failure. A “cured” patient was a pulmonary TB patient with bacteriologically confirmed TB at treatment initiation who was smear- or culture-negative in the last month of treatment and on at least one previous occasion. The category “completed treatment” included TB patients who completed treatment without evidence of failure but with no recorded negative sputum smear or culture results in the last month of treatment and on at least one previous occasion because either the tests were not performed or the results were not available [17].

This study was conducted in accordance with the standards defined by the Declaration of Helsinki. The protocol was approved by the Ethics Committee of the Public Health School at the University of Kinshasa (Permit Number: ESP/CE/042/2015). This was a retrospective study. Therefore, consent was not obtained, and patient records were anonymized and de-identified prior to analysis. The ethics committee approved this procedure.

All the collected data were verified for completeness and entered into Excel before being transferred to SPSS version 20.0 for analysis. Frequencies and averages are reported for the variables of interest. The chi-square test was performed to evaluate the associations between variables in the bivariate analysis. A multivariate logistic regression was performed to identify predictors of treatment outcome based on the adjusted odds ratio (aOR) and 95% confidence interval (CI). A p-value <0.05 was considered statistically significant.

## Results

**Fig 2:**
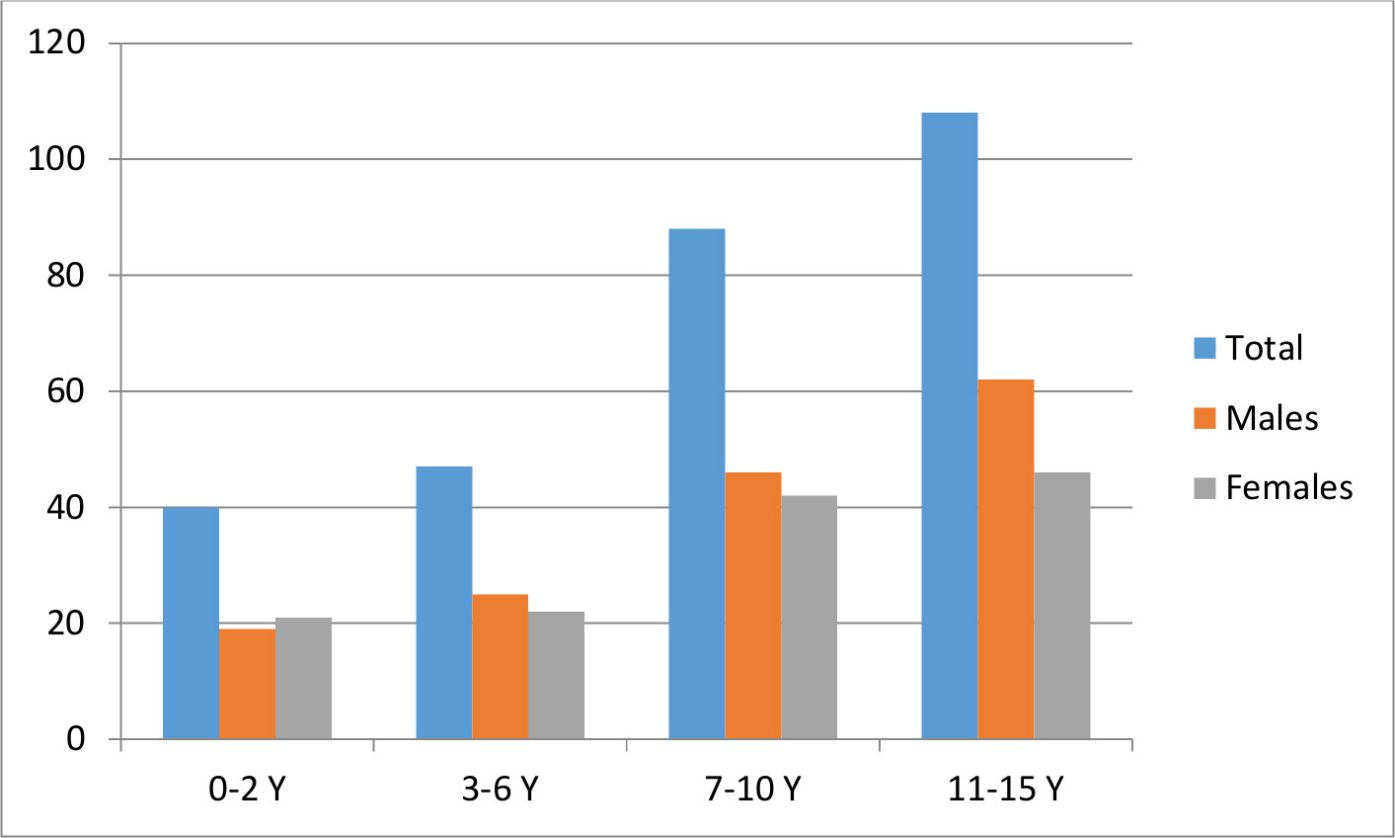
Distribution of patients by age and sex.

During the study period, 16,621 children were admitted to the pediatric department, 291 (1.7%) were diagnosed with TB, and only 283 were included in this study. Eight children were excluded based on a lack of complete data or missing charts. Among the included children, the sex ratio was 1.16 in favor of boys, and the populations by age group were as follows: 0 to 2 years, 40 (14.1%); 3 to 6 years, 47 (16.6%); 7 to 10 years, 88 (31.1%); and 11 to 15 years, 108 (38.2%) (Fig 2). The average age was 8.7 (± 4.4) years. The household size was categorized into 3 groups (<4 persons, 4–6 persons and ˃6 persons), and there were 23 (8.1%) families in the first group, 144 (50.9%) in the second group, and 116 (41.0%) in the third group. In 43 cases (15.1%), we identified a history of TB contact (Table 1).

**Table 1:** Demographic, clinical, and outcome data and paraclinical characteristics according to gender.

Characteristic	Total n (%)	Male n (%)	Female n (%)	p-value
**Age (years)**	283 (100)	152 (53.5)	131 (46.5)	0.43
0–2	40 (14.1)	18 (6.3)	22 (7.8)	
3–6	47 (16.6)	29 (10.2)	18 (6.4)	
7–10	88 (31.1)	49 (17.3)	39 (13.9)	
11–15	108 (38.2)	56 (19.7)	52 (18.4)	
**Household size**	283 (100)	152 (53.5)	131 (46.5)	0.15
<4	23 (8.1)	8 (2.8)	15 (5.3)	
4–6	144 (50.9)	81 (28.5)	63 (22.3)	
˃6	116 (41.0)	63 (22.2)	53 (18.8)	
**Patient residence**	283 (100)	152 (53.5)	131 (46.5)	0.45
Near UCK^*^	228 (80.6)	120 (42.4)	108 (38.1)	
Far from UCK^¶^	55 (19.4)	32 (11.3)	23 (8.1)	
**Reported TB contact** (n = 43 or 15.2%)	43 (100)	24 (55.8)	19 (44.2)	0.76
**Type of TB**	283 (100)	152 (54.0)	131 (46.0)	**0.04**
PTB+	40 (14.1)	18 (11.8)	22 (16.8)	
PTB-	82 (29.0)	53 (34.9)	29 (22.1)	
EPTB	159 (56.2)	81 (53.3)	78 (59.5)	
MDR-TB	2 (0.7)	0 (0.0)	2 (1.5)	
**Patient category**	283 (100)	152 (53.5)	131 (46.5)	0.29
Category 1	86 (30.4)	46 (16.2)	40 (14.1))	
Category 2	5 (1.8)	4 (1.3)	1 (0.3)	
Category 3	190 (67.1)	102 (36.0)	88 (31.1)	
Category 4	2 (0.7)	0 (0.0)	2 (0.7)	
**Nutritional status** (n = 170 or 60.0%)	170 (100)	93 (54.7)	77 (45.3)	0.79
No malnutrition	108 (63.6)	57 (33.5)	51 (30.0)	
Moderate malnutrition	29 (17.1)	17 (10.0)	12 (7.1)	
Severe malnutrition	33 (19.4)	19 (11.2)	14 (8.2)	
**HIV status** (n = 72 or 25.4%)	283 (100)	152 (53.7)	131 (46.3)	0.49
Positive	7 (2.5)	4 (1.4)	3 (1.1)	
Negative	65 (23.0)	39 (13.7)	26 (9.2)	
Unknown	211 (74.5)	109 (38.4)	102 (36.0)	
**Ziehl-Neelsen test** (n = 247 or 87.3%)	247 (100)	132 (53.4)	115 (46.6)	0.17
Positive	39 (15.8)	17 (6.9)	22 (8.9)	
Negative	208 (84.2)	115 (46.5)	93 (37.7)	
**Lowenstein culture** (n = 97 or 34.3%)	97 (100)	54 (55.7)	43 (44.3)	0.25
Positive	32 (33.0)	19 (19.6)	13 (13.4)	
Negative	65 (67.0)	35 (36.1)	30 (30.9)	
**Chest X-ray** (n = 173 or 61.1%)	173 (100)	89 (51.4)	84 (48.6)	**0.016**
Pathologic	144 (83.2)	80 (46.2)	64 (37.0)	
Normal	29 (16.8)	9 (5.2)	20 (11.6)	

TB: tuberculosis; PTB+: positive pulmonary TB sputum smear; PTB-: negative pulmonary TB sputum smear; EPTB: extra-pulmonary tuberculosis

^*^Near UCK: patients who lived in the same neighborhood as the UCK

^¶^ Far from UCK: patients who lived outside the UCK neighborhood.

The majority of patients (183; 64.6%) lived in the UCK neighborhood. The patients who lived in more distant areas were transferred to receive the best care. However, only 247 (87.3%) patients underwent AFB testing by the Ziehl-Neelsen method (Ziehl-Neelsen positivity rate, 16%; 39 positive results); 97 (34.2%) patients underwent culture and drug sensitivity testing (DST) in solid milieu, and 34 (35%) of the results were positive.

Based on the diagnostic results, 82 (29.0%) patients had PTB-, 40 (14.1%) presented with PTB+, 159 (56.2%) had EPTB, and 2 (0.7%) had MDR-TB, among whom 1 (0.3%) had no positivity for *Mycobacterium tuberculosis*. The EPTB cases included 118 (74.2%) cases of peripheral lymphadenitis TB, 11 (6.9%) cases of pleural effusion TB, 8 (5.0%) cases of meningitis TB, 6 (3.8%) cases of vertebral TB, 5 (3.1%) cases of abdominal TB, 4 (2.5%) cases of miliary TB and pericardial TB, and 3 (1.9%) cases of osteoarticular TB. Regarding therapeutic categorization, 86 (30.4%) children were in category 1, of whom 5 (1.8%) were retreated; 190 (67.1%) were in category 3; and 2 (0.7%) were in category 4. The age, household size, patient residence, type of TB, therapeutic category, nutritional status, HIV status, Ziehl-Neelsen test results, Lowenstein culture and chest X-ray results are presented according to sex. The associations between sex and chest X-ray (p = 0.016) and type of TB (p = 0.047) were significant (Table 1).

Some adverse effects were reported during treatment. Anemia occurred in 17 (6.0%) patients, among whom 3 (1.0%) required a blood transfusion; 4 (14.0%) patients had a moderate elevation of uric acid, and 32 (11.3%) had slightly high transaminase levels between the second and fifth months of treatment. There were no reported cases of neurological or visual disorders. One patient presented with a cutaneous reaction during the first week of treatment. Without any investigation, treatment was stopped and then progressively reintroduced over 2 weeks with a good response. The HIV status of 72 patients was known (25.4%; 2.5% positive).

After follow-up and therapeutic evaluation, 167 children (59.0%) had completed treatment, 30 (10.6%) were cured, and 7 (2.4%) had failed, necessitating a change in the current regimen. Four (1.4%) of the patients died; 1 patient on SLD died after treatment failure, whereas 3 patients died during a category 1 regimen because of respiratory distress, severe anemia and septic shock. One category 2 patient was lost to follow-up but returned 5 months later in a state of septic and hypovolemic shock. A total of 20 (7.0%) patients were declared defaulters, and 55 (19.4%) were referred to a health facility nearest their home. The results are presented in Tables 1 and 2. There were significant independent associations between TB outcome and the type of TB (p = 0.000), Ziehl-Neelsen test (p = 0.000), Lowenstein culture (p = 0.004), HIV status (p = 0.05), and chest X-ray (p = 0.05) (Table 2). Based on the logistic regression, none of these factors was a significant predictor of a poor outcome. However, the Ziehl-Neelsen results, nutritional status and reported TB contact remained in the model (Table 3).

**Table 2:** Factors associated with patient outcome.

Characteristic	Cured n = 30 (10.6%)	Completed treatment n = 167 (59.0%)	Failed n = 7 (2.4%)	Defaulted n = 20 (7.0%)	Transferred^ⱡ^ n = 55 (19.4%)	Died n = 4 (1.4%)	p-value
**Reported TB contact**	10	23	2	3	5	0	**0.04**
**(n = 43)**							
**Age (years)**	30	167	7	20	55	4	0.09
0–2	7 (23.3)	21 (12.6)	1 (14.3)	4 (20.0)	7 (12.7)	0 (0.0)	
3–6	0 (0.0)	33 (19.8)	1 (14.3)	2 (10.0)	9 (16.4)	2 (50.0)	
7–10	6 (20.0)	53 (31.7)	0 (0.0)	8 (40.0)	20 (36.4)	1 (25.0)	
11–15	17 (56.7)	60 (35.9)	5 (71.4)	6 (30.0)	19 (34.5)	1 (25.0)	
**Type of TB**	30	167	7	20	55	4	**0.000**
PTB+	29 (96.7)	3 (1.8)	3 (42.9)	0 (0.0)	5 (9.1)	0 (0.0)	
PTB-	1 (3.3)	55 (32.9)	1 (14.3)	7 (35.0)	17 (30.9)	1 (25.0)	
EPTB	0 (0.0)	111 (65.3)	2 (28.6)	13 (65.0)	33 (60.0)	2 (50.0)	
MDR-TB	0 (0.0)	0 (0.0)	1 (14.3)	0 (0.0)	0 (0.0)	1 (25.5)	
**Form of EPTB (n = 159)**	0 (0.0)	111 (69.8)	2 (1.2)	12 (7.5)	32 (10.1)	2 (1.2)	0.095
Peripheral lymphadenitis	0 (0.0)	80 (67.8)	1 (0.8)	9 (7.6)	28 (23.7)	0 (0.0)	
Pleural effusion	0 (0.0)	9 (81.8)	0 (0.0)	0 (0.0)	2 (18.1)	0 (0.0)	
Meningitis TB	0 (0.0)	4 (50.0)	1 (12.5)	1 (12.5)	0 (0.0)	2 (25.0)	
Vertebral TB	0 (0.0)	5 (83.3)	0 (0.0)	1 (16.6)	0 (0.0)	0 (0.0)	
Abdominal TB	0 (0.0)	3 (60.0)	0 (0.0)	0 (0.0)	2 (40.0)	0 (0.0)	
Miliary TB	0 (0.0)	4 (100)	0 (0.0)	0 (0.0)	0 (0.0)	0 (0.0)	
Pericardial TB	0 (0.0)	4 (100)	0 (0.0)	0 (0.0)	0 (0.0)	0 (0.0)	
Osteoarticular TB	0 (0.0)	2 (69.8)	0 (0.0)	1 (33.3)	0 (0.0)	0 (0.0)	
**Nutritional status (n = 170)**	13	103	2	14	35	3	0.94
No malnutrition	7 (53.8)	69 (67.0)	1 (50.0)	9 (64.3)	21 (60.0)	1 (33.3)	
Moderate malnutrition	3 (23.1)	16 (15.5)	0 (0.0)	2 (14.3)	7 (20.0)	1 (33.3)	
Severe malnutrition	3 (23.1)	18 (17.5)	1 (50.0)	3 (21.4)	7 (20.0)	1 (33.3)	
**HIV status (n = 72)**	9	42	2	3	15	1	**0.05**
Positive	2 (22.2)	2 (4.8)	1 (50.0)	0 (0.0)	1 (6.7)	1 (100)	
Negative	7 (77.8)	40 (95.2)	1 (50.0)	3 (100)	14 (93.3)	0 (0.0)	
**Ziehl-Neelsen test (n = 247)**	30	142	7	18	46	4	**0.000**
Positive	30 (100)	1 (0.7)	2 (28.6)	0 (0.0)	5 (10.9)	1 (25.0)	
Negative	0 (0.0)	141 (99.3)	5 (71.4)	18 (100)	41 (89.1)	3 (75.0)	
**Lowenstein (n = 97)**	19	50	6	7	13	2	**0.004**
Positive	14 (73.7)	9 (18.0)	3 (50.0)	1 (14.3)	5 (38.5)	2 (100)	
Negative	5 (26.3)	41 (82.0)	3 (50.0)	6 (85.7)	8 (61.5)	0 (0.0)	
**Chest X-ray (n = 173)**	30	95	5	10	30	3	**0.05**
Pathologic	30 (100)	73 (76.8)	5 (100)	8 (80.0)	26 (86.7)	2 (66.7)	
Normal	0 (0.0)	22 (23.2)	0 (0.0)	2 (20.0)	4 (13.3)	1 (33.3)	

**ⱡ** Patients referred to the health center nearest their home

TB: tuberculosis; PTB+: positive pulmonary TB sputum smear; PTB-: negative pulmonary TB sputum smear; EPTB: extra-pulmonary tuberculosis.

**Table 3:** Predictors of treatment outcome. Peu ou pas d’éléments présomptifs

Characteristic	Treatment success^*^ n (%)	Poor treatment outcome^¥^ n (%)	OR (95% CI)
**Ziehl-Neelsen**^**ⱡ**^ **positivity**	31 (12.55)	3 (1.21)	2.35 (0.25–21.89)
**Reported TB contact**	33 (11.66)	5 (1.06)	3.05 (0.27–33.95)
**Malnutrition**^**€**^	40 (23.52)	8 (4.70)	2.17 (0.17–27.66)

We note only the factors that remained at the 10th step of the logistic regression model. Transferred patients are not represented in this table because they do not belong to either of these two groups.

*Treatment success: cured and completed treatment

**¥** Poor treatment outcome: failed, defaulted, died.

**ⱡ** Only 247 patients underwent Ziehl-Neelsen testing.

**€** Nutritional status was assessed only for children aged 0 to 10 years (n = 170).

## Discussion

This study focused on describing childhood TB at a tertiary hospital in the DRC, and it reflects the general situation of TB support for children in our area because the national TB policy is applied in the same way throughout the country and at all levels of the health system.

The TB prevalence is high in our country; therefore, bacille Calmette–Guérin (BCG) vaccination is obligatory after birth. However, this vaccination has insufficient efficacy, and new vaccinations are needed [18]. We can also attribute this high prevalence to poor TB control in adults; additionally, other contributing factors, such as poor socioeconomic status and malnutrition, are very common in our country.

The distribution of patients by age group was unusual in this study [10]; there were few cases in children aged less than 2 years (14.1%). Many hypotheses potentially support this observation. Younger children are immediately treated in a primary health care facility after they are clinically diagnosed with TB, and older children are transferred to a referral care facility for diagnosis before treatment. In some cases, older children die of pneumonia and are therefore missed as tuberculosis cases. The diagnostic difficulties regarding younger children, particularly the difficulty in obtaining expectorate samples [19, 20] or gastric aspirates [21, 22], may also explain this result. Due to these diagnostic difficulties, new tools are needed to improve TB diagnosis in children [23–26]. The low sensitivity and specificity of scoring systems [27] and the infrequency with which chest radiographs are obtained may also contribute to this result. Therefore, there is a need to conduct a multicenter study in the DRC to confirm whether the observations presented herein are generally applicable or result from hospital bias. Reported TB contact is an important factor in childhood TB infection [28] because it increases the risk for TB [29]; this factor was significantly associated with outcome in the present study. We found a low TB-HIV co-infection rate (2.5%), perhaps because HIV status was not known for all the patients. TB detection methods must be better optimized [30], and the guidelines concerning the integration of TB and HIV treatment in pediatric populations must be improved if we want to achieve successful outcomes [31].

In this study, a majority of cases were EPTB and PTB-, which is an unusual distribution. The WHO reported that TB without evidence of *Mycobacterium* is widespread among children, with a frequency of 13% for all ages in 2012 [1]. Some authors from limited-resource countries have reported the same results as ours, which they hypothesized to result from limited resources for investigations, the greater frequency of the pauci-bacillary form of TB among children, and the difficulty with expectoration [32, 33]. However, other authors in Africa [34–36] reported lower rates of EPTB. The high rate of EPTB reported in the present study can be attributed to the under-diagnosis of pulmonary TB because of diagnostic difficulties and the limited number of patients who were able to realize a chest X-ray. Additionally, EPTB was likely over-diagnosed because of the low sensitivity of the scoring system and diagnostic difficulty [37]; thus, children with EPTB are more readily referred to a tertiary facility than those with PTB. Alternatively, perhaps there are inherent biases in a hospital study.

Identifying the adverse effects associated with anti-TB drugs was not the primary objective of this study. However, the results suggested that the treatments were generally well tolerated. We cannot attribute the minor adverse effects to only TB medication because there was not a control group to assess whether particular symptoms, for example anemia, was due to other causes, such as malaria or sickle cell anemia. The treatment of children with TB is well codified in the DRC according to WHO guidance [38]. It is detailed in a support guide for TB entitled “PATI” (TB Program integrated into basic health care or primary health care), which is in its 4^th^ edition since 2008 [13]; the 5^th^ edition is in press and includes the new WHO recommendations [17]. This support guide facilitates the monitoring of patients undergoing TB treatment who are referred to the primary health facility nearest their home. The present research is one of few studies that have described the support available for children with TB in our country [21, 39–41]; although the problem of TB in infants is not recent [42], the global plan to stop TB 2011–2015 is working to solve this problem [43]. Unfortunately, several bottlenecks have made it difficult to achieve these objectives.

In fact, the health organization of the DRC is based on the strategy of primary health care as defined by the WHO, with each health area serving approximately 100,000 to 300,000 inhabitants. The financial barriers to health care services, the lack of information systems for monitoring, the lack of human resources and laboratory capacity, and the absence of a mutual health insurance system constitute obstacles to the achievement of millennium development goals.

Despite these difficulties, 70% of patients completed their treatment at UCK; this rate was higher than that reported in Botswana [36] or Malawi [35] but lower than that in other studies in Africa that showed higher treatment success rates [34, 44]. In fact, 10.8% of our patients failed defaulted, or died, suggesting poor case management and an inadequate control program for childhood TB. A study conducted in Nigeria reported a poor treatment outcome in 10.8% of TB/HIV-negative children, similar to the result in our study [45]. In southern Ethiopia [46], another study reported a high rate of poor outcomes (18%). In southwestern Iran, 8.3% of children experienced a poor treatment outcome [47]; this rate was lower than that in our study. In Denmark, the rate of overall treatment success from 2001 to 2009 was 93.8%. Only one child (˂0.1%) died [48]. In the United States, a review of 145 adolescents showed that 92% of patients recovered without complication [49]. A similar result was obtained in Vietnam, with 93% treatment success, likely because long hospital stays allow patients to complete a substantial portion of the intensive treatment phase with optimal adherence and because the low rate of drug resistance evokes better therapeutic responses [33]. Interventions to increase treatment adherence for pediatric TB have proven to be effective based on improved treatment success rates in low- and middle-income countries [50]. Developing countries must deal with new challenges in the management of childhood TB [51, 52].

Five factors were associated with patient outcome: type of TB, HIV status, positive Ziehl-Neelsen test, positive Lowenstein culture and pathologic chest X-ray. In Ethiopia, Hailu et al identified 4 factors (age, type of TB, category and HIV co-infection) that were associated with TB outcome [53]. In Iran, Alavi et al found that age, low body weight, household contact and exposure to cigarette smoke were the main risk factors for poor treatment outcome [47]. The results of a study conducted on South African MDR-TB and HIV co-infected children revealed that malnutrition and severe radiographic findings were associated with an unfavorable outcome [54].

The factors that are the same regardless of study site may require more attention when monitoring children with TB. In the present study, all of these factors were not adequately evaluated at the participating institution. The priorities must be good integration and practice of national recommendations, even in tertiary facilities, and more attention must be given to children presenting with TB associated with these factors.

Although this study is one of few in this field to be conducted in this country, there were some limitations. A multicenter prospective study is necessary because it eliminates biases inherent in a retrospective hospital study, such as the present study. Particularly unusual findings, such as the age and TB form distributions and the low number of files with radiographic findings, must be further evaluated in future studies. There is also a need to perform additional studies to explore other TB susceptibility factors and other factors related to treatment outcome [55–56].

## Conclusions

TB treatment in children is facilitated by the implementation of a DOTS program. This investigation revealed that the support remains insufficient in our context; this fact justifies the development of alternative diagnostic techniques and the provision of increased accessibility to all diagnostic tools. Reported TB contacts, nutritional status, type of TB, co-infection with HIV, Ziehl-Neelsen positivity, Lowenstein culture and chest X-rays may require more attention when monitoring childhood TB. Others studies can be performed to assess other factors, such as genetic factors, that are associated with TB outcome in Congolese children.
